# Normal life expectancy after all-trans retinoic acid and arsenic trioxide for acute promyelocytic leukemia

**DOI:** 10.1038/s41375-026-02989-0

**Published:** 2026-06-08

**Authors:** A. Piciocchi, A. Talone, M. Messina, P. de Fabritiis, G. Marsili, V. Sargentini, S. Soddu, L. Cicconi, L. Guarnera, M. D. Divona, P. Musto, F. Ferrara, S. D’Ardia, E. Borlenghi, F. Buccisano, M. Breccia, F. Efficace, P. Fazi, A. Venditti, F. Lo Coco, U. Platzbecker, M. Vignetti, M. T. Voso

**Affiliations:** 1 Gruppo Italiano Malattie Ematologiche dell’Adulto (GIMEMA) Foundation, Rome, Italy; 2 Department of Hematology, Santa Maria Goretti Hospital, Polo Universitario Pontino, Latina, Italy; 3 Hematology, Department of Biomedicine and Prevention, University Tor Vergata, Rome, Italy; 4 Unit of Hematology and Stem Cell Transplantation, AOUC Policlinico and Department of Precision and Regenerative Medicine and Ionian Area, Aldo Moro University School of Medicine, Bari, Italy; 5 Ematologia, AORN A. Cardarelli, Napoli, Italy; 6 SC Hematology, AOU Città della Salute e della Scienza di Torino, Turin, Italy; 7 SC di Ematologia, ASST Spedali Civili, Brescia, Italy; 8 Hematology, Department of Translational and Precision Medicine, Az. Policlinico Umberto I-Sapienza University, Rome, Italy; 9 Department of Hematology, Oncology and Stem cell Transplantation, University Hospital, Leipzig, Germany

**Keywords:** Acute myeloid leukaemia, Epidemiology

## To the Editor:

Acute promyelocytic leukemia (APL) has undergone one of the most profound therapeutic transformations in hematologic oncology. The first clear clinical and morphological description of the disease, provided in 1957 by MacMahon and Forman, characterized APL as a distinct and highly fatal form of acute leukemia, marked by its fulminant clinical course and severe coagulopathy [1]. Their early observations established the fundamental clinical profile of APL and highlighted the urgent need for treatments capable of counteracting its catastrophic hemorrhagic complications and rapid progression. For several decades, therapeutic improvements occurred incrementally. The introduction of anthracycline-based chemotherapy represented a meaningful step forward, improving remission rates and short-term survival [2]. However, chemotherapy alone was limited by considerable toxicity, treatment-related mortality, and a persistent risk of relapse. A paradigm shift occurred with the discovery of all-trans retinoic acid (ATRA) and its ability to induce terminal differentiation of leukemic promyelocytes through direct targeting of the PML::RARA oncoprotein [3]. This breakthrough introduced the concept of differentiation therapy and transformed APL from one of the most lethal acute leukemias into the most curable subtype of acute myeloid leukemia. A subsequent transformative milestone was the recognition of the potent synergy between ATRA and arsenic trioxide (ATO), another agent targeting PML::RARA. ATO was shown to promote degradation of the fusion protein and effectively eradicate leukemic stem cells. The pivotal 2013 trial by Lo Coco et al. demonstrated that a chemotherapy-free regimen of ATRA plus ATO was superior to ATRA combined with chemotherapy in low- to intermediate-risk APL, offering higher cure rates with remarkably reduced toxicity [4]. This established ATRA/ATO as the preferred frontline therapy for most patients. More recently, the large-scale Harmony APL project confirmed the broad applicability of ATRA/ATO, demonstrating excellent outcomes regardless of age or Sanz risk score [5]. In parallel, the APOLLO Trial expanded the evidence base by demonstrating the impressive efficacy of ATRA/ATO in high-risk APL as well, further consolidating this combination as the cornerstone of modern APL treatment [6]. Alongside survival, attention increasingly shifted toward patients’ long-term well-being. The work by Efficace et al. provides key evidence in this regard, showing that individuals treated with ATRA/ATO within the APL0406 trial reported better long-term quality-of-life outcomes compared to those treated with ATRA combined with chemotherapy [7]. These results underscore that modern chemotherapy-free regimens not only ensure prolonged survival but also preserve long-term quality of life. Despite these therapeutic achievements, early death—often occurring before the disease is recognized or before APL-specific therapy can be initiated—remains the main barrier to improving population-level outcomes. Moreover, although long-term survival is excellent among patients who complete therapy, whether ATRA/ATO-treated survivors achieve a life expectancy equivalent to that of the general population has not been conclusively demonstrated. To address this question, we analyzed mortality patterns of patients treated with ATRA/ATO within the GIMEMA APL0406 and APL0618 studies, comparing observed deaths with those expected in an age-, sex-, and region-matched Italian reference population. Data were obtained from two GIMEMA studies adopting a frontline chemotherapy-free approach for newly diagnosed acute promyelocytic leukemia (APL). From the APL0406 randomized trial, only patients assigned to the experimental arm receiving all-trans retinoic acid (ATRA) in combination with arsenic trioxide (ATO) were included (EudraCT 2006-006188-22; ClinicalTrials.gov NCT00482833) [8]. The APL0618 study contributed an observational cohort including patients treated uniformly with ATRA/ATO in routine practice (ClinicalTrials.gov NCT03751917) [9]. Written informed consent was obtained from all participants prior to inclusion in the studies.

The primary objective was to assess whether long-term survivors treated with ATRA/ATO experienced mortality similar to that expected in the general population. For each patient, follow-up accrued from the end of therapy to death or last known assessment. Expected mortality was derived using age-, sex- and region-specific life tables from the Italian National Institute of Statistics (ISTAT). By applying these reference mortality rates to the person-years accumulated by the study population, the expected number of deaths was estimated for a population with demographic characteristics analogous to those of the cohorts examined. To address demographic imbalances between the study cohorts and the Italian reference population, indirect standardization was employed. The comparison between observed and expected mortality was expressed as a standardized mortality ratio (SMR), accompanied by its 95% confidence interval, calculated through Poisson-based methods appropriate for analyses with low event counts [10]. Geographic variation in baseline mortality was accounted for by stratification into three predefined macro-areas of residence (North, Center, South/Islands). A pooled analysis was conducted to provide an integrated estimate of long-term mortality among patients treated with frontline ATRA/ATO.

The study population consisted of consecutive patients with low- to intermediate-risk APL enrolled in two GIMEMA studies and treated with frontline ATRA/ATO. Eligibility criteria were consistent with standard diagnostic requirements for APL, including molecular confirmation of the PML::RARA fusion gene. A total of 249 patients met the inclusion criteria, comprising 129 individuals treated in the chemotherapy-free ATRA/ATO arm of the APL0406 randomized trial between 2007 and 2013 and 120 enrolled in the APL0618 observational study treated between 2019 and 2023. The cohort included 119 men and 130 women, with a median age of 50 years, and was geographically representative of the Italian population, with 117 patients residing in Northern Italy, 38 in Central Italy and 94 in Southern Italy or the Islands. Follow-up duration was substantial across both studies and allowed a reliable assessment of late mortality; the median follow-up was 5.6 and 2.8 years for APL0406 and APL0618, respectively.

During long-term observation, two deaths occurred in the APL0406 ATRA/ATO cohort (1 colorectal cancer and 1 infection) and two relapses at 22 and 27 months; two deaths also occurred in the APL0618 cohort (1 hemorrhage and 1 lung cancer) and 1 relapse after 14 months. When age-, sex- and region-specific mortality rates from national life tables were applied to the person-years accumulated by each study population, the number of expected deaths closely approximated that observed. In APL0406, the standardized mortality ratio was 0.89 (95% CI: 0.10 to 3.22), indicating a mortality pattern consistent with that expected for the general population. In APL0618, the standardized mortality ratio was 1.17, (95%CI: 0.14 to 4.21), again showing no deviation from the mortality anticipated for a demographically matched reference population.

**Fig. 1:**
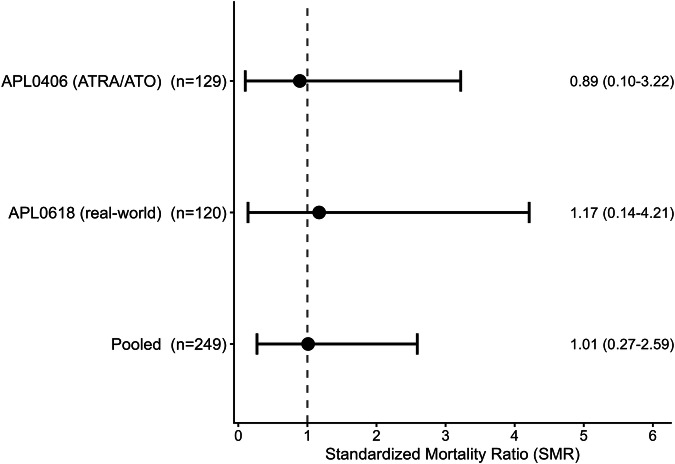
Standardized mortality ratios (SMR) with corresponding 95% confidence intervals for patients treated with frontline ATRA and arsenic trioxide in the experimental arm of the GIMEMA APL0406 randomized trial, in the observational GIMEMA APL0618 cohort, and in the pooled analysis. The vertical reference line indicates the expected mortality in the age-, sex- and region-matched Italian general population.

In the combined dataset, four deaths were documented, essentially mirroring the 3.95 deaths predicted on the basis of age, sex, geographic distribution and follow-up time. The resulting standardized mortality ratio for the pooled cohort was 1.01 (95%CI: 0.27 to 2.59), confirming the absence of any measurable excess mortality among long-term survivors treated with ATRA and ATO. These results, including the estimates for each cohort and for the integrated analysis, are illustrated in Fig. 1. This study provides a comprehensive assessment of long-term mortality among patients with APL treated exclusively with the modern, chemotherapy-free ATRA/ATO regimen across two independent GIMEMA trials. By comparing observed deaths with those expected in a matched Italian reference population, we sought to determine whether treatment advances have translated into a normalization of life expectancy. Although part of the analysis derives from patients enrolled in a randomized clinical trial, the inclusion of the observational APL0618 cohort—reflecting routine clinical practice—improves the generalizability of the findings. Our findings demonstrate that patients who complete ATRA/ATO therapy exhibit overall mortality essentially identical to that predicted for the general population, as reflected by a pooled SMR of 1.01. This indicates that APL survivors treated with this regimen do not experience measurable excess mortality and can anticipate a long-term prognosis equivalent to individuals without hematologic malignancies. Relapse after frontline ATRA/ATO was also extremely uncommon, consistent with previously reported long-term results of the APL0406 study. Placed in historical context, this outcome is extraordinary. APL was long regarded as the most lethal variant of acute leukemia, primarily due to its rapid onset and life-threatening coagulopathy. Subsequent therapeutic progress with anthracycline-based chemotherapy improved remission rates, but long-term toxicity and relapse remained significant concerns. The introduction of ATRA revolutionized treatment by enabling differentiation of leukemic blasts, while the addition of ATO eliminated the need for cytotoxic chemotherapy for most patients and drastically improved cure rates [3–7]. Despite these achievements, uncertainty persisted regarding potential late mortality, particularly in light of findings in other leukemia subtypes, where survivors face increased risks of secondary malignancies, cardiovascular complications, or therapy-related toxicities [11]. Our results offer strong reassurance that such late effects are minimal after ATRA/ATO therapy. The exceptionally low number of observed deaths and the near-unity SMR suggest negligible disease activity, limited treatment-related toxicity, and no evidence of delayed mortality risk. Quality-of-life data provide an essential dimension to the interpretation of these findings. Analyses by Efficace et al. from the APL0406 trial have shown that, even over the long term (after a median time since diagnosis of 8 years), the health status profile of patients with APL previously treated with ATRA/ATO is broadly comparable to that of their peers in the general population [12]. These data point to sustained long-term benefits, from the patient’s perspective, associated with this treatment. This alignment of objective survival measures with patient-reported outcomes underscores the unique success of modern APL therapy. It must be emphasized, however, that these favorable long-term outcomes apply to patients who survive the initial, critical phase of the disease. Early mortality—largely due to hemorrhagic complications, diagnostic delays, or failure to initiate ATRA promptly—remains the most significant challenge in APL management. Given that virtually all long-term survivors can anticipate normal life expectancy, reducing early death rates represents the most important avenue for improving overall survival at the population level. Strategies focused on rapid recognition of APL, immediate administration of ATRA even when diagnosis is only suspected, and optimized supportive care are therefore essential. This study has limitations that warrant consideration. The low number of deaths, while reassuring clinically, results in wide confidence intervals around SMR estimates. For the same reason, the statistical power to detect small differences in mortality compared with the general population is limited. Consequently, the confidence intervals around the SMR estimates are wide. However, the close agreement between observed and expected deaths suggests that any potential excess mortality, if present, is likely to be small and clinically negligible. Nevertheless, the consistency between two independent cohorts, the standardized methodology, and the alignment with quality-of-life data support the reliability of our conclusions. Future analyses based on larger international datasets with extended follow-up would provide more precise mortality estimates and allow evaluation of potential subgroups at risk. In conclusion, our results demonstrate that patients with APL treated with frontline ATRA/ATO who survive the acute phase achieve long-term survival indistinguishable from that of the general Italian population. These findings confirm that the modern therapeutic paradigm for APL not only cures the disease but fully restores normal life. Further progress in APL outcomes will depend primarily on efforts to reduce early mortality through rapid diagnosis, early ATRA administration, and optimized supportive care.
